# Safety of live, attenuated oral vaccines in HIV-infected Zambian adults: Oral vaccines in HIV

**DOI:** 10.1016/j.vaccine.2012.06.079

**Published:** 2012-08-17

**Authors:** Rose Banda, Vera Yambayamba, Bwalya Daka Lalusha, Edford Sinkala, Melissa Chola Kapulu, Paul Kelly

**Affiliations:** a Tropical Gastroenterology & Nutrition Group, Department of Medicine, University of Zambia School of Medicine, University Teaching Hospital, Lusaka, Zambia; b Blizard Institute of Cell and Molecular Science, Barts & The London School of Medicine and Dentistry, Queen Mary University of London, Turner Street, London E1 2AD, UK

**Keywords:** HIV, Oral vaccines, Rotavirus vaccine, Typhoid vaccine, ETEC vaccine, Phase 1 study

## Abstract

**Background:**

Current recommendations are that HIV-infected persons should not be given live vaccines. We set out to assess potential toxicity of three live, attenuated oral vaccines (against rotavirus, typhoid and ETEC) in a phase 1 study.

**Methods:**

Two commercially available oral vaccines against rotavirus (Rotarix) and typhoid (Vivotif) and one candidate vaccine against Enterotoxigenic *Escherichia coli* (ACAM2017) were given to HIV seropositive (*n* = 42) and HIV seronegative (*n* = 59) adults. Gastrointestinal symptoms were sought actively by weekly interview up to 1 month of vaccination. In rotavirus vaccine recipients, intestinal biopsies were collected by endoscopy and evaluated for expression of IL-8 and pro-inflammatory cytokines.

**Results:**

No difference was observed between symptoms in HIV infected and HIV uninfected vaccinees, except for diarrhoea reported more than 7 days after the last dose of vaccine. If only diarrhoea episodes within 7 days of vaccination are included, diarrhoea was not more frequent in HIV seropositive than in HIV seronegative vaccinees (OR 6.7, 95% CI 1.2–67; *P* = 0.09). However, if later episodes of diarrhoea are included, a significant increase in diarrhoea was demonstrated (OR 5.3, 95% CI 0.98–53; *P* = 0.04). All episodes were mild and transient. IL-8 was slowly up-regulated over the week following vaccination (*P* = 0.02), but IL-β, IFNγ or TNFα were not.

**Conclusions:**

No evidence was found of adverse events following administration of these three vaccines, except for late episodes of diarrhoea which may not be attributable to vaccination. Our data do not support the need for a prohibition on oral administration of live, attenuated vaccines to all HIV infected adults, though further work on severely immunocompromised adults and children are required.

## Highlights

► Current practice is to avoid live, attenuated vaccines in HIV infection. ► Three live, attenuated oral vaccines were given to Zambian adults. ► We found no evidence of adverse events related to HIV infection. ► The recommendation to avoid live oral vaccines may need to be re-examined.

## 1 Introduction

Diarrhoeal disease remains one of the commonest causes of death in children, especially in the malnourished. Up to 2 million children die of diarrhoea each year, and diarrhoea has effects on long-term development and growth [1,2]. In Zambia, the prevalence of diarrhoea in children under 5 years of age is very high, with 21.2% of mothers reporting diarrhoea in a 2-week period [3]. Diarrhoea in children is mostly attributed to rotavirus and Enterotoxigenic *Escherichia coli* (ETEC). The prospects for global provision of adequate quantities of clean water are as distant as ever, with probably over 1 billion people unable to access safe drinking water. Vaccines against rotavirus, cholera and typhoid are available, but some are live, attenuated vaccines which would need to be used in populations with high HIV prevalence. It would also be desirable to offer protection against diarrhoea-causing pathogens to HIV infected adults and children, so it is imperative to determine if these vaccines are safe in HIV infected individuals.

Recent highly authoritative guidelines [4] on prevention of opportunistic infection in HIV-infected children recognise the potential risk of giving live, inactivated vaccines to potentially immunocompromised children but make it clear that few data are available on the magnitude of that risk. A recent analysis of rotavirus in relation to HIV, and the experience of a trial in South Africa in which HIV infected children were given a rotavirus vaccine, do suggest that it is safe [5]. The oral live, attenuated cholera vaccine CVD103HgR was found to be safe in HIV-infected adults in Mali [6], and there is evidence that oral polio vaccine is safe in HIV infected children [7]. However, uncertainty remains due to the paucity of data in African populations [8,9].

In order to address these concerns we analysed our experience of giving any of three live, attenuated vaccines to Zambian adults. Both the bacterial vaccines are known to be sensitive to ciprofloxacin and so we were confident that this evaluation was safe in the carefully monitored setting in which they were given. In the event, none of the recipients needed any medical support or antibiotic treatment. As rotavirus vaccination programmes are rolled out across sub-Saharan Africa, it is important to assess the potential toxicity of this vaccine in HIV infection, so a subset of participants receiving the rotavirus vaccine underwent intestinal biopsy to evaluate expression of IL-8, IL-β, IFNγ and TNFα.

## 2 Methods

### 2.1 Participants

The study was conducted in Lusaka, Zambia, between February 2008 and October 2009. Participants were drawn from the Misisi cohort, a mixed cohort of HIV seropositive and seronegative adults, which is defined only by residence in a defined area [10]. Potential participants were not given vaccines if they were pregnant or lactating, had experienced diarrhoea within 1 month before their planned participation, had taken antibiotics or non-steroidal anti-inflammatory drugs in the same period, had been vaccinated with any other vaccine within 6 months, or were found to have infection with an intestinal helminth by examination of 3 stool samples taken over a 3–5-day period. Ethical approval was obtained from the University of Zambia Research Ethics Committee (007-10-07) and all participants gave written informed consent.

### 2.2 Vaccine administration

Rotarix (Glaxo Smith Kline, Brentford, UK) is derived from a human rotavirus which was attenuated by repeated passage and is safe in children [11]. The second vaccine, ACAM2017 (Acambis plc, Cambridge, UK), was derived from a spontaneous LT-negative ETEC isolate which has deletions of the chorismate synthase gene *aroC*, the membrane proteins *ompC* and *ompF*, and the toxin genes for LT, ST and EAST. The gene for CS1 [12] has been added and it induces specific mucosal IgA against coli surface (CS) antigens CS1, CS2 and CS3 [13]. The third vaccine, Vivotif (Ty21a vaccine; Berna Biotech, Bern, Switzerland), is the only licensed oral typhoid vaccine [14]. The gene *galE* is inactivated and it is unable to express the pathogenicity factor Vi; it has an excellent safety record. Vivotif is immunogenic in the host but has low environmental survival. Vivotif induces anti-LPS systemic IgG and IgA, antibody secreting cells in peripheral blood, and cell mediated immunity (CMI). Immunity conferred by Ty21a lasts up to 7 years but uniquely requires 3–4 doses given only 1–2 days apart [15]. Rotarix was administered as a single dose in 1.3 ml liquid carbonate buffer according to the manufacturer's instructions. ACAM2017 was administered as a suspension of bacteria prepared by Acambis plc as previously described [13]. The dose of viable organisms in the vaccine vials (3 × 10^10^) was confirmed in the laboratory in three test vials which were discarded in order to avoid contamination of the vials used for vaccination. The dose contained in each vial was administered as one dose and the vial was then discarded. Vivotif was administered as a capsule, initially as a single dose in 23 participants, then 2 doses (on days 1 and 3) in 5 participants, then 3 doses (the full dosing schedule on days 1, 3, and 5) in a further 5 participants, then the full schedule for the remainder of the study when safety and acceptability concerns had been allayed. Altogether, 81 participants received Vivotif.

### 2.3 Adverse event recording

Each participant was interviewed at 7, 14, 21 and 28 days after vaccine administration. Direct questions were asked about the experience of the symptoms listed in Table 2. Full blood count data collected prior to vaccine administration and either 7 or 14 days afterwards were also compared.

### 2.4 Intestinal cytokine expression

Jejunal biopsies were collected endoscopically using an Olympus SIF-10 endoscope under diazepam sedation as previously described [16–18]. Biopsies were obtained 1 day prior to vaccine administration and at 1 (*n* = 4), 2 (*n* = 6), 4 (*n* = 6), or 7 (*n* = 5) days after the first vaccine dose. Each participant underwent two endoscopies and these biopsies were evaluated as a before/after pair. Biopsies were collected into 200 μl Tri Reagent (Sigma, Poole, UK) and snap-frozen in liquid nitrogen followed by storage at −80 °C. Biopsies were used within 3 months, and RNA isolated as previously described [18]. Following reverse transcription, real time quantitative polymerase chain reaction (RT-qPCR) was carried out using SYBR Green enzyme buffer (Qiagen) with primers shown in Table 1 for the following cytokines: interleukin (IL)-8, IL-1β, interferon (IFN)-γ, and tumour necrosis factor (TNF)-α.

### 2.5 Data analysis

Diarrhoea or other AEs attributable to vaccine were considered if the onset was within 7 days of the last dose of vaccine. All AEs were compared in HIV seropositive versus HIV seronegative participants and proportions analysed using Fisher's exact test. Cytokine mRNA measurements were normalised to GAPDH and expressed as -fold change from baseline to post-vaccination sample, and statistical significance evaluated using the Wilcoxon signed-rank test to determine if there was a significant change in gene expression following vaccination. For IL-8 mRNA, where a temporal trend was apparent following vaccination, linear regression of mRNA and time was used to confirm statistical significance.

## 3 Results

Between February 2008 and October 2009, 100 participants between the ages of 18 and 60 years were randomly allocated to receive one of the three vaccines: Rotarix (*n* = 24), ETEC (*n* = 21) or Vivotif (*n* = 81), or to act as controls who received no vaccine (*n* = 21). Forty-seven of these participants who were available were subsequently invited to participate on a second occasion, either as vaccinee or control, at time points separated by intervals of at least 1 year. No vaccinee received the same vaccine twice. Demographic and clinical characteristics of the participants are shown in Table 2. Altogether, 34 HIV seropositive adults received 58 courses of live, attenuated vaccines orally at one time point or another. Vaccinees and controls were well matched for sex, age, body mass index, and (in the HIV seropositives) CD4 count (Table 2).

### 3.1 Clinical adverse events

Diarrhoea was reported within 7 days of the last dose of vaccine by 6 participants, all of whom had received 3 doses of Vivotif and 5 of whom were HIV seropositive (OR for HIV seropositivity 6.3, 95% CI 0.67–303; *P* = 0.09). The intervals after which these were experienced were 3, 4, 4, 8, 10, and 13 days after the first dose. None of these had diarrhoea which they judged to have been serious enough to seek treatment but two had taken the day off work. The CD4 counts of those HIV seropositive participants who experienced diarrhoea within 7 days of last vaccine administration were (in ascending order) 175, 179, 351, 670, and 845 cells/μl.

If the period of attribution is extended to 28 days after the first dose of vaccine, 11 episodes of diarrhoea were reported by 10 vaccinees. Of these, 3 were within 7 days, 5 between 8 and 14 days, 2 between 15 and 21 days, and 1 between 22 and 28 days. Of the 10 vaccinees who experienced diarrhoea, 8 were HIV seropositive (Table 3). The two HIV seronegative vaccinees reported diarrhoea 13 days after Vivotif and 21 days after ACAM2017. Including these later episodes of diarrhoea changes the Odds Ratio for HIV seropositivity to 5.3 (95% CI 0.98–53; *P* = 0.04).

Abdominal pain was reported by 3 vaccine recipients. In two of these instances, pain occurred during diarrhoeal illnesses, with onset 4 and 10 days after the first doses of Vivotif. One participant reported pain without diarrhoea 5 days after the first dose of Vivotif.

Fever (subjective, not confirmed) was reported by one HIV negative man the day after rotavirus vaccination, and by two HIV positive men 13 and 16 days after ETEC vaccination, respectively. None of these participants sought medical care.

Loss of appetite (scoring 1 on analogue scale of 1–10) was reported only by one HIV seronegative participant within 24 h of receiving ACAM2017. Three other HIV positive participants reported loss of appetite, but all over 3 weeks after the vaccine dose and designated not attributable. Only one HIV seronegative participant reported nausea or vomiting, and that was 12 days after a dose of Vivotif.

### 3.2 Intestinal cytokine expression

**Fig. 1:**
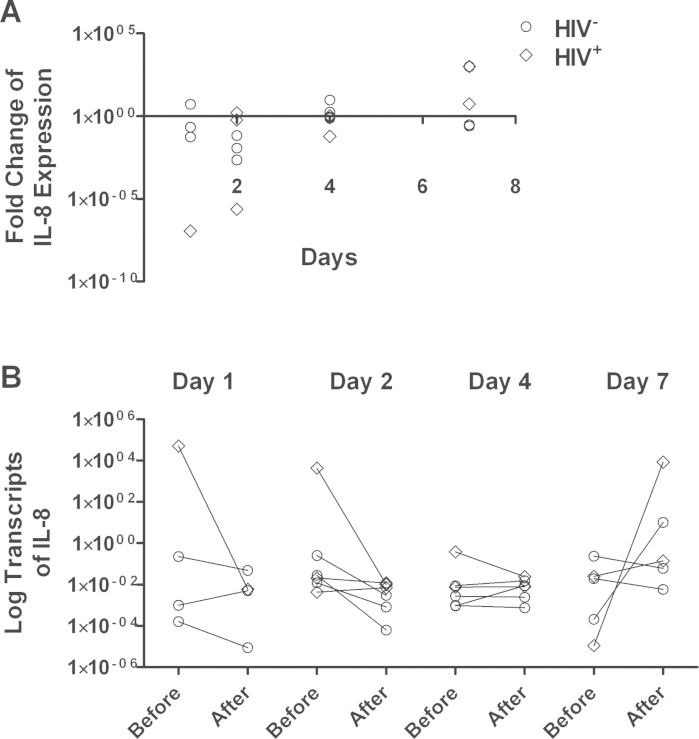
Changes in IL-8 mRNA expression 1, 2, 4, or 7 days after rotavirus vaccine administration. (A) Each point represents the -fold change in mRNA transcript abundance (relative to GAPDH) from the baseline value to the value obtained in the biopsy taken at that time point. Each individual participant was biopsied twice (baseline and at the time point shown) so each point represents all the information obtained from one participant. These individual changes are shown in panel (B). The change over time in IL-8 mRNA shows a statistically significant trend (*P* = 0.02) using linear regression. Circles represent HIV seronegative individuals and diamonds HIV seropositive individuals.

**Fig. 2:**
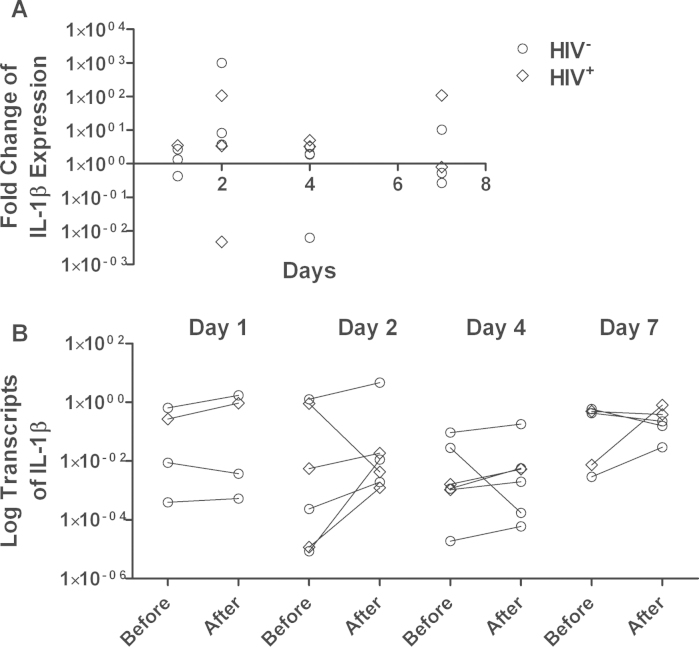
Changes in IL-1β mRNA expression 1, 2, 4, or 7 days after rotavirus vaccine administration. (A) Each point represents the -fold change in mRNA transcript abundance (relative to GAPDH) from the baseline value to the value obtained in the biopsy taken at that time point. Each individual participant was biopsied twice (baseline and at the time point shown) so each point represents all the information obtained from one participant. These individual changes are shown in panel (B).

**Fig. 3:**
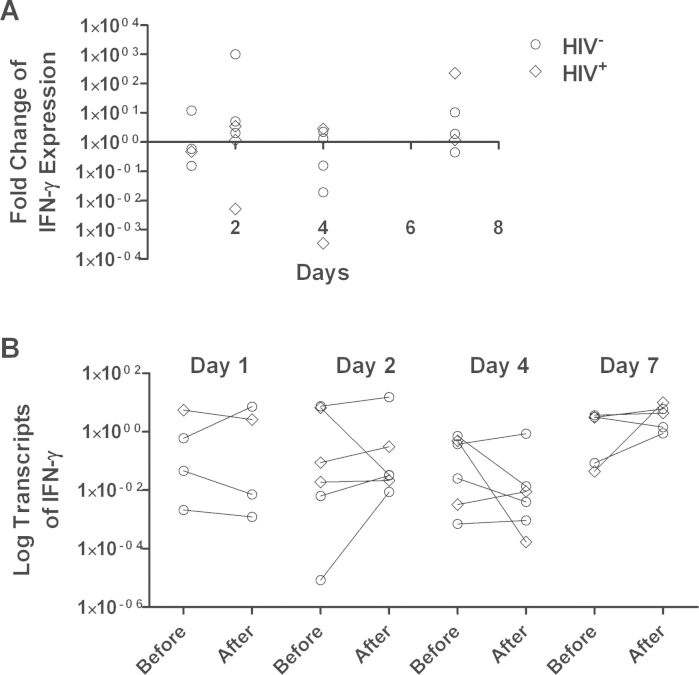
Changes in interferon-γ mRNA expression 1, 2, 4, or 7 days after rotavirus vaccine administration. (A) Each point represents the -fold change in mRNA transcript abundance (relative to GAPDH) from the baseline value to the value obtained in the biopsy taken at that time point. Each individual participant was biopsied twice (baseline and at the time point shown) so each point represents all the information obtained from one participant. These individual changes are shown in panel (B).

All recipients of the rotavirus vaccine underwent enteroscopy for jejunal biopsy collection, but RT-PCR results did not meet quality standards for two samples, and one participant did not return on the scheduled day, so data are available for 21 participants. By RT-qPCR, mRNA of IL-8 showed an immediate down-regulation followed by a slow up-regulation which was statistically significant (*P* = 0.02) in a regression model against time (Fig. 1). There was no discernible effect of vaccination on IL-1β (Fig. 2) or IFNγ (Fig. 3). TNFα expression was undetectable in a considerable number of samples: in 6 cases there was no detectable expression before or after vaccination; in 5 cases mRNA was detected only before vaccination, and in 5 cases only after vaccination. In the remaining 5 cases, there was a modest down-regulation, but this was not statistically significant in view of the small number of data pairs. HIV-infected participants did not differ from HIV-uninfected participants with respect to changes in cytokine expression following vaccination, and those biopsies in which TNFα expression was not detectable were not more likely to come from HIV-infected participants (data not shown).

## 4 Discussion

The safety of live, attenuated vaccines in HIV infected people is of paramount importance if vaccines are to play any role in reducing the burden of common diseases in tropical populations. In this study we found that in 34 HIV seropositive adults given a total of 58 courses of three live, attenuated oral vaccines there was no evidence of serious adverse events: no hospitalisations, no episodes of diarrhoea requiring treatment, no significant febrile illnesses, and no increase in symptoms such as abdominal pain, nausea or loss of appetite. There was no evidence of haematological toxicity. If we accept that oral vaccines do not cause diarrhoea after 7 days have elapsed beyond the final dose of vaccine, there was no increase in diarrhoea. The interpretation of diarrhoea data in this setting is difficult if we use HIV seronegative adults as the comparison group, as at any given point in time HIV infected adults have a higher incidence rate of diarrhoeal disease [19]. We believe that this explains the higher diarrhoea incidence after 7 days following vaccination. Our data are compatible with the hypothesis that these vaccines lead to a modest increase in mild, transient episodes of diarrhoea beyond 1 week in HIV infected adults. They are also explicable with there being a consistently increased risk of diarrhoea in HIV throughout the period of observation.

We found no evidence that vaccines induce intestinal inflammation. IL-8 is a chemokine expressed by epithelial cells on contact with potentially invasive bacteria. The other, pro-inflammatory, cytokines showed no change in expression over the week following vaccination. While these data do not rule out a pathogenic effect of these vaccines, they offer considerable reassurance that rotavirus vaccine does not induce inflammation. We are not aware of other studies which have examined intestinal tissue following vaccination against which we can compare our data. Wild-type rotavirus infection leads to significant mucosal inflammation and although this inflammatory response is not fully characterised in humans, there is evidence that at least interferon-γ is implicated in the systemic response [20]. In cell culture models using rat and human cells, TNFα, IFN-β and IL-6 were induced by rotavirus dsRNA [21]. In animal models, an early IL-8 response is seen [22]. Our data are surprising in as much as the IL-8 response was delayed, appearing to rise from an initial down-regulation, for up to 7 days.

The participants we enrolled were drawn from a community cohort study where most HIV infected adults have been offered, and agree to, monitoring in an HIV treatment programme, and take HAART where necessary. Only 6 of our participants had CD4 counts below 200 cells/μl, all of whom had experienced a rapid drop in CD4 count from their previous clinic visit. Thus we cannot be confident that these vaccines are safe in adults with severe immunodeficiency (although the bacterial strains are sensitive to ciprofloxacin and could be easily treated if symptoms develop). For certain infections, parenteral vaccines are available (such as the Vi polysaccharide vaccine for typhoid) or oral killed vaccines (such as the killed whole-cell cholera vaccine which has been shown to be safe in an outbreak in Mozambique [23]). However, oral administration of live, attenuated vaccines combines the advantage of ease of administration on a large scale with good immunogenicity, at least over 2–3 years, and these vaccines remain attractive for further development. While our findings need to be confirmed in larger studies, they do suggest that safety may not be an obstacle to exploiting the potential for oral vaccination in southern Africa, and we do not support the view [9] that live oral vaccines should be withheld from all HIV-infected adults. However, further studies are needed of vaccine safety in severely immunocompromised adults and children.

## Conflicts of interest

The authors have no commercial or other associations which might pose a conflict of interest. The funding agency played no part in the collection of data, analysis, or preparation of the manuscript.

## Figures and Tables

**Table 1:** Primers used for quantitative PCR for intestinal cytokine expression.

	Forward (5′ to 3′)	Reverse (3′ to 5′)
IL-1β	CGGCCACATTTGCTAAGA	AGGGAAGCGGTTGCTCATC
TNFα	CCCAGGCAGTCAGATCATCTTC	GCTTGAGGGTTTGCTACAACATG
IFNγ	ACTGACTTGAATGTCCAACGCA	ATCTGACTCCTTTTTCGCTTCC
IL-8	TGTGTGTAAACATGACTTCCAAGCT	GCAAAACTGCACCTTCACACAG
GAPDH	CCAGCCGAGCCACATCGCTC	ATGAGCCCCAGCCTTCTCCAT

**Table 2:** Demographic and clinical characteristics of vaccine recipients.

	Rotavirus vaccine (*n* = 24)	ETEC vaccine (*n* = 21)	Typhoid vaccine (*n* = 81)	Control (*n* = 21)	*P*
Sex (M:F)	10:14	11:10	29:52	7:14	0.52
Age (yrs; median, IQR)	35 (29–41)	42 (35–55)	39 (29–47)	42 (30–50)	0.37
HIV infected (*n*, %)	9 (38%)	7 (33%)	39 (48%)	11 (52%)	0.36
BMI (kg/m^2^) (median, IQR)	22.3 (19.3–26.9)	21.6 (19–24.6)	23.0 (19.26.3)	20.4 (19.2–24.6)	0.66
CD4 count^a^ (median, IQR)	417 (294–576)	380 (292–402)^b^	374 (245–545)^c^	406 (294–541)	0.29
CD4 count ≤ 200 cells/μl	0	0	6	1	

*P* values refer to results of statistical testing across all groups.

a CD4 count expressed as cells/μl.

b Data only available for 6 samples.

c Data only available for 38 samples.

**Table 3:** Reported adverse events in Zambian adults given live, attenuated oral vaccines.

	Rotavirus		ETEC		Vivotif		Total vaccinated		*P*
	HIV+ (*n* = 9)	HIV− (*n* = 15)	HIV+ (*n* = 7)	HIV− (*n* = 14)	HIV+ (*n* = 42)	HIV− (*n* = 39)	HIV+ (*n* = 58)	HIV− (*n* = 68)	
Diarrhoea (within 7 days of last dose of vaccine)	0	0	0	0	5	1	5	1	0.09
Diarrhoea (within 28 days of first dose of vaccine)	0	0	3	1	6^a^	1	9	2	0.04
Abdominal pain	0	0	0	0	3	0	3	0	0.09
Fever	0	1	0	0	0	0	0	1	1.00
Loss of appetite	0	0	0	1	0	0	0	1	1.00
Nausea/vomiting	0	0	0	0	0	0	0	0	–
Fall in Hb > 1 g/dl	3	2	0	3	4	3	7	8	1.00
Fall in WBC >1 × 10^9^/l	0	3	1	0	7	7	8	10	1.00

The *P* value shown relates to HIV seropositive vs. seronegative participants (i.e. not including the second episode of diarrhoea in that vaccinee who experienced two episodes) in the total group of those vaccinated, using Fisher's exact test.

a Two of these episodes were in the same vaccinee.
